# Supplementary material for: No Evidence for Classic Thrombotic Microangiopathy in COVID-19
Source: J Clin Med. 2021 Feb 9;10(4):671. doi: 10.3390/jcm10040671 (PMC7916239; doi:10.3390/jcm10040671)
Supplement: Supplementary file 1 [file jcm-10-00671-s001.pdf]

**Figure S1:** Organ damage markers in patients with COVID-19

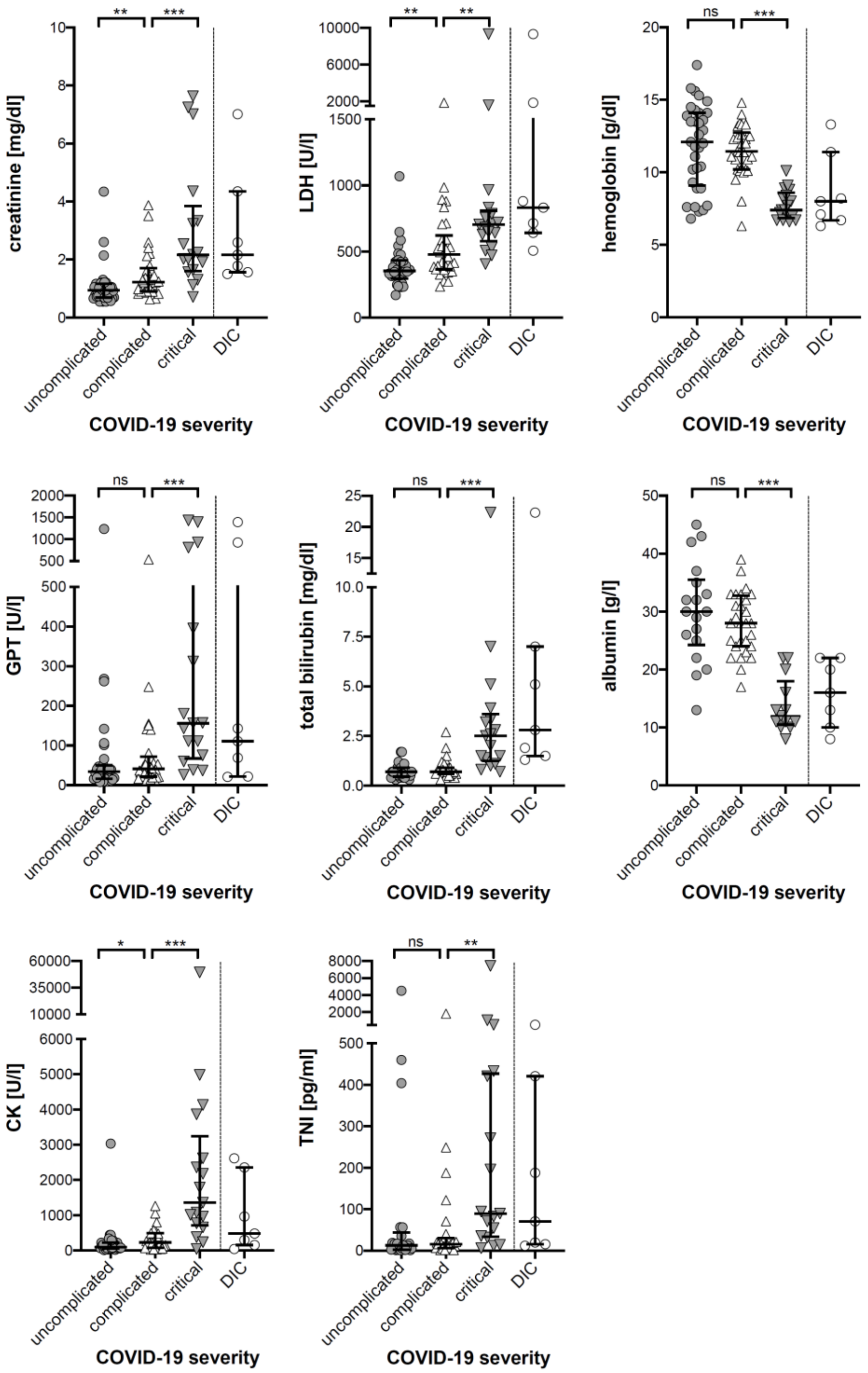


Highest values for creatinine, lactate dehydrogenase (LDH), alanine-aminotransferase (GPT), total bilirubin, creatine kinase (CK) and troponin I (TNI) and the lowest values for hemoglobin and albumin are presented as indicated. The markers for organ damage are plotted against the severity of coronavirus disease-2019 (COVID-19) and the occurrence of DIC, respectively. Medians and interquartile ranges are provided. Comparison between subgroups were based on Mann-Whitney-U-test (ns, not significant, * $p < 0.05$, ** $p < 0.01$, *** $p < 0.001$).

**Table S1.1**: Comparison of the 65 patients positive for SARS-CoV-2 included (Table 1), and the 20 excluded for analysis of hemostatic alterations because of lacking laboratory data.

| Characteristics | total | included | excluded |
|---|---|---|---|
| Number | 85 | 65 | 20 |
| Age (years) (IQR) | 66 (53-79) | 69 (57-79) | 54 (39-76) |
| Sex (male/female) | 47/38 (55.3/44.7%) | 41/24 (63.1/36.9%) | 14/6 (70.0/30.0%) |
| BMI (kg/m²) (IQR) [a] | 27.1 (23.7-33.5) | 27.2 (24.2-33.6) | 27.1 (21.5-31.8) |
| **Preexisting comorbidities** | | | |
| Arterial Hypertension | 44 (51.8%) | 38 (58.5%) | 6 (30.0%) |
| Diabetes mellitus | 16 (18.8%) | 14 (21.5%) | 2 (10.0%) |
| Obesity (BMI ≥ 30kg/m²) [a] | 30/79 (38.0%) | 23/62 (37.1%) | 7/17 (41.2%) |
| Chronic respiratory disease | 13 (15.3%) | 11 (16.9%) | 2 (10.0%) |
| Cardiovascular disease | 21 (24.7%) | 17 (26.2%) | 4 (20.0%) |
| Cerebrovascular disease | 15 (17.6%) | 11 (16.9%) | 4 (20.0%) |
| **Preexisting anticoagulation** | | | |
| DOAC | 1 (1.2%) | 1 (1.5%) | 0 (0.0%) |
| Vitamin K antagonist | 1 (1.2%) | 1 (1.5%) | 0 (0.0%) |
| Platelet aggregation inhibitors | 23 (27.1%) | 19 (29.2%) | 4 (20.0%) |
| **Clinical complications during COVID-19 infection** | | | |
| Thromboembolic events | 4 (4.7%) | 4 (6.2%) | 0 (0%) |
| **Medical care during COVID-19 infection** | | | |
| Hospitalized | 72 (84.7%) | 63 (96.9%) | 9 (45.0%) |
| Outpatients | 13 (15.3%) | 2 (3.1%) | 11 (55.0%) |
| Intensive care | 20 (23.5%) | 20 (30.8%) | 0 (0.0%) |
| Oxygen supplementation | 48 (56.5%) | 46 (70.8%) | 2 (10.0%) |
| Invasive ventilation | 17 (20%) | 17 (26.2%) | 0 (0.0%) |
| **Anticoagulation during COVID-19 infection** | | | |
| None | 22 (25.9%) | 7 (10.8%) | 15 (75.0%) |
| Prophylactic dose LMWH | 48 (56.5%) | 43 (66.2%) | 5 (25.0%) |
| Therapeutic dose LMWH | 14 (16.5%) | 14 (21.5%) | 0 (0.0%) |
| **Clinical outcome of COVID-19 infection** | | | |
| Uncomplicated | 37 (43.5) | 19 (29.2%) | 18 (90.0%) |
| Complicated | 31 (36.5%) | 29 (44.6%) | 2 (10.0%) |
| Critical | 17 (20.0%) | 17 (26.2%) | 0 (0.0%) |
| Deceased | 10 (11.8%) | 9 (13.8%) | 1 (5.0%) |

Patient characteristics are presented as median (interquartile range) or number (%). BMI, body mass index; COVID-19, coronavirus disease-2019; DOAC, direct oral anticoagulants; IQR, interquartile range; LMWH, low molecular weight heparin.

[a] BMI and obesity could only be determined in 79 of 85 patients due to missing anthropometric data.

**Table S1.2**: Comparison of patient characteristics between groups of COVID-19 severity

| Characteristics | Total patients | COVID-19 uncomplicated versus complicated | COVID-19 complicated versus critical | no DIC versus DIC |
|---|---|---|---|---|
| **Age (years) (IQR)** | 69 (57-79) | 0.137 [b] | 0.189 [b] | 0.560 [b] |
| **Sex (male/female)** | 41/24 (63.1/36.9%) | 0.683 [d] | 0.097 [d] | **0.041** [e] |
| **BMI (kg/m²) (IQR)** [a] | 27.2 (24.2-33.6) | 0.553 [c] | 0.881 [c] | 1.000 [c] |
| **Preexisting comorbidities** | | | | |
| **Arterial hypertension** | 38 (58.5%) | 0.135 [d] | 0.534 [d] | 0.228 [e] |
| **Diabetes mellitus** | 14 (21.5%) | 0.065 [e] | 0.908 [d] | 0.683 [e] |
| **Obesity (BMI ≥30kg/m²)** [a] | 23/62 (37.1%) | 0.373 [d] | 0.301 [d] | 0.243 [e] |
| **Chronic respiratory disease** | 11 (16.9%) | 0.451 [e] | 1.000 [e] | 1.000 [e] |
| **Cardiovascular disease** | 17 (26.2%) | 0.725 [d] | 0.739 [e] | 0.385 [e] |
| **Cerebrovascular disease** | 11 (16.9%) | 1.000 [e] | 0.058 [e] | 1.000 [e] |
| **Terminal renal insufficiency** | 2 (3.1%) | n.d. | n.d. | n.d. |
| **Venous thromboembolic history** | 4 (6.2%) | 1.000 [e] | 0.135 [e] | 0.373 [e] |
| **Pulmonary embolism** | 1 (1.5%) | n.d. | n.d. | n.d. |
| **Deep vein thrombosis** | 4 (6.2%) | 1.000 [e] | 0.135 [e] | 0.373 [e] |
| **Preexisting anticoagulation** | | | | |
| **DOAC** | 1 (1.5%) | n.d. | n.d. | n.d. |
| **Vitamin K antagonist** | 1 (1.5%) | n.d. | n.d. | n.d. |
| **Platelet aggregation inhibitors** | 19 (29.2%) | 0.396 [e] | **0.045** [e] | 0.096 [e] |
| **Clinical complications during COVID-19** | | | | |
| **Renal failure** | | | | |
| **AKIN 1** | 3 (4.6%) | n.d. | n.d. | n.d. |
| **AKIN 3** | 15 (23.1%) | 0.267 [d] | **<0.0001** [d] | **0.044** [e] |
| **Thromboembolic events** | 4 (6.2%) | 0.396 [e] | **0.045** [e] | 1.000 [e] |
| **Acute coronary syndrome** | 3 (4.6%) | 0.512 [e] | 1.000 [e] | 0.294 [e] |
| **Myocarditis** | 2 (3.1%) | 0.152 [e] | n.d. | 1.000 [e] |
| **Medical care during COVID-19** | | | | |
| **Hospitalized** | 63 (96.9%) | n.d. | n.d. | n.d. |
| **Intensive care** | 20 (30.8%) | 0.267 [e] | **<0.0001**[d] | **0.025** [e] |
| **Oxygen supplementation** | 46 (70.8%) | **<0.0001** [d] | n.d. | n.d. |
| **Invasive ventilation** | 17 (26.2%) | n.d. | **<0.0001** [d] | 0.070 [e] |
| **Renal replacement therapy** | 10 (15.4%) | 1.000 [e] | n.d. | n.d. |
| **Anticoagulation during COVID-19** | | | | |
| **None** | 7 (10.8%) | 0.097 [e] | 0.524 [e] | 0.568 [e] |
| **Prophylactic dose LMWH** | 43 (66.2%) | 0.164 [e] | **<0.0001** [d] | **0.039** [e] |
| **Therapeutic dose LMWH** | 14 (21.5%) | 1.000 [e] | **<0.0001** [d] | 0.164 [e] |
| **DOAC** | 1 (1.5%) | n.d. | n.d. | n.d. |
| **Clinical outcome of COVID-19** | | | | |
| **Deceased** | 9 (13.8%) | 0.211 [e] | 0.387 [e] | 0.131 [e] |

Patient characteristics as median (interquartile range) or number (%). AKIN, AKIN Classification for Acute Kidney Injury; BMI, body mass index; COVID-19, coronavirus disease-2019; DOAC, direct oral anticoagulants; DIC, disseminated intravascular coagulopathy; IQR, interquartile range; LMWH, low molecular weight heparin.

[a] BMI and obesity could only be determined in 62 of 65 patients due to missing anthropometric data.

[b] t-test, [c] Mann-Whitney-U-test , [d] Chi-squared test, [e] Fishers exact test.

**Table S2.1:** Rates of abnormal laboratory values of 65 patients with COVID-19 and analyzed for hemostatic abnormalities.

| Parameter | total | | Uncomplicated COVID-19 | | Complicated COVID-19 | | Critical COVID-19 | | DIC [a] | |
|---|---|---|---|---|---|---|---|---|---|---|
| **Number of patients** | 65 | # | 19 | # | 29 | # | 17 | # | 7 | # |
| **LDH elevated (>245 U/l)** | 60 (95.2%) | 63 | 15 (88.2%) | 17 | 28 (96.6%) | 29 | 17 (100.0%) | 17 | 7 (100.0%) | 7 |
| **AST elevated (>31/ 35 U/l) [b]** | 56 (88.9%) | 63 | 14 (73.7%) | 19 | 26 (92.9%) | 28 | 16 (100.0%) | 16 | 6 (100.0%) | 6 |
| **ALT elevated (≥35/ 50U/l) [b]** | 33 (51.6%) | 64 | 5 (27.8%) | 18 | 14 (48.3%) | 29 | 14 (82.4%) | 17 | 5 (71.4%) | 7 |
| **GGT elevated (>36/ 64 U/l) [b]** | 45 (71.4%) | 63 | 12 (66.7%) | 18 | 17 (60.7%) | 28 | 16 (94.1%) | 17 | 5 (71.4%) | 7 |
| **Total bilirubin elevated (> 1.2mg/dl)** | 17 (26.6%) | 64 | 0 (0.0%) | 18 | 4 (13.8%) | 29 | 13 (76.5%) | 17 | 7 (100.0%) | 7 |
| **Albumin decreased (<28 g/l)** | 31 (53.4%) | 58 | 6 (35.3%) | 17 | 12 (42.9%) | 28 | 13 (100.0%) | 13 | 7 (100.0%) | 7 |
| **CK elevated (>170/ 200 U/l) [b]** | 40 (64.5%) | 62 | 9 (47.4%) | 19 | 15 (57.7%) | 26 | 16 (94.1%) | 17 | 5 (71.4%) | 7 |
| **TNI elevated (>24 pg/ml)** | 27 (44.3%) | 61 | 7 (36.8%) | 19 | 6 (24.0%) | 25 | 14 (82.4%) | 17 | 4 (57.5%) | 7 |
| **Creatinine elevated (>1.02/ 1.18 mg/dl) [b]** | 38 (59.4%) | 64 | 6 (31.6%) | 19 | 17 (60.7%) | 28 | 15 (88.2%) | 17 | 7 (100.0%) | 7 |
| **Hemoglobin decreased (<12/ 13.5 g/dl) [b]** | 52 (80%) | 65 | 12 (63.25) | 19 | 23 (79.3%) | 29 | 17 (100.0%) | 17 | 7 (100.0%) | 7 |
| **Platelet count (<150/nl)** | 22 (33.8%) | 65 | 5 (26.3%) | 19 | 11 (37.9%) | 29 | 6 (35.3%) | 17 | 5 (71.4%) | 7 |
| **Platelet count (<50/nl)** | 3 (4.6%) | 65 | 0 (0.0%) | 19 | 1 (3.4%) | 29 | 2 (11.8%) | 17 | 2 (28.6%) | 7 |
| **CRP elevated (>5 mg/l)** | 64 (98.5%) | 65 | 18 (94.7%) | 19 | 29 (100.0%) | 29 | 17 (100.0%) | 17 | 7 (100.0%) | 7 |
| **PCT elevated (>0.5 ng/ml)** | 22 (33.8%) | 65 | 1 (5.3%) | 19 | 5 (17.3%) | 29 | 16 (94.1%) | 17 | 6 (85.7%) | 7 |
| **INR elevated (>1.25)** | 32 (49.2%) | 65 | 6 (31.6%) | 19 | 9 (31.0%) | 29 | 17 (100.0%) | 17 | 7 (100.0%) | 7 |
| **Fibrinogen elevated (>390 mg/dl)** | 58 (100.0%) | 58 | 16 (100.0%) | 16 | 25 (100.0%) | 25 | 17 (100.0%) | 17 | 7 (100.0%) | 7 |
| **Fibrinogen decreased (<100 mg/dl)** | 0 (0.0%) | 58 | 0 (0.0%) | 16 | 0 (0.0%) | 25 | 0 (0.0%) | 17 | 0 (0.0%) | 7 |
| **D-dimer elevated (>0.5 mg/l)** | 55 (84.6%) | 65 | 14 (73.7%) | 19 | 24 (85.7%) | 29 | 17 (100.0%) | 17 | 7 (100.0%) | 7 |
| **D-dimer elevated (>2 mg/l)** | 25 (39.7%) | 65 | 4 (21.1%) | 19 | 8 (28.6%) | 29 | 13 (81.3%) | 17 | 6 (85.7%) | 7 |

Number and rates (%) of laborytory values above (elevated) or below (decreased) the limits of normal or indicated cut-offs, respectively. The laboratory values were obtained during the follow up until 15th May 2020. CK, creatine kinase; COVID-19, coronavirus disease-2019; CRP, C-reactive protein; DIC, disseminated intravascular coagulopathy; AST, aspartate-aminotransferase; ALT, alanine-aminotransferase; GGT, gamma-glutamyltransferase; INR, international normalized ratio; LDH, lactate dehydrogenase; PCT, procalcitonin; TNI , troponin I.

[#] Number of total patients and patients with uncomplicated, complicated, and critical course of COVID-19, and with DIC for whom laboratory values were available.

[a] Patients with DIC are a subset of patients with complicated and critical COVID-19.

[b] Reference values in brackets for women/men are indicated.

**Table S2.2**: Comparison of laboratory parameters between groups of COVID-19 severity

| Parameter | Total patients | | COVID-19 uncomplicated versus complicated | COVID-19 complicated versus critical | no DIC versus DIC |
|---|---|---|---|---|---|
| **Number of patients** | 65 | # | p-Value | p-Value | p-Value |
| **LDH$^{max}$ (U/l)** | 507 (381-705) | 63 | 0.090 [c] | **0.004** [c] | **0.001** [c] |
| **LDH elevated (>245 U/l)** | 60 (95.2%) | 63 | 0.545 [e] | 1.000 [e] | 1.000 [e] |
| **AST$^{max}$ (U/l)** | 76 (46-187) | 63 | 0.104 [c] | **0.001** [c] | 0.077 [c] |
| **AST elevated (>31/ 35 U/l)** [a] | 56 (88.9%) | 63 | 0.102 [e] | 0.526 [e] | 1.000 [e] |
| **ALT$^{max}$ (U/l)** | 51 (33-1429) | 64 | 0.743 [c] | **0.001** [c] | 0.260 [c] |
| **ALT elevated (≥35/ 50U/l)** [a] | 33 (51.6%) | 64 | 0.164 [d] | **0.022** [d] | 0.428 [e] |
| **GGT$^{max}$ (U/l)** | 87 (43-180) | 63 | 0.848 [c] | **<0.0001** [c] | 0.316 [c] |
| **GGT elevated (>36/ 64 U/l)** [a] | 45 (71.4%) | 63 | 0.683 [d] | **0.017** [e] | 1.000 [e] |
| **Total bilirubin$^{max}$ (mg/dl)** | 0.8 (0.6-1.4) | 64 | 0.400 [c] | **<0.0001** [c] | **<0.0001** [c] |
| **Total bilirubin elevated (>1.2mg/dl)** | 17 (26.6%) | 64 | 0.283 [e] | **< 0.0001** [d] | **<0.0001** [e] |
| **Albumin$^{min}$ (g/l)** | 26 (20-32) | 58 | 0.290 [b] | **<0.0001** [b] | **0.002** [b] |
| **Albumin decreased (<28 g/l)** | 31 (53.4%) | 58 | 0.616 [d] | **<0.0001** [d] | 0.120 [e] |
| **CK$^{max}$ (U/l)** | 301 (97-798) | 62 | 0.421 [c] | **<0.0001** [c] | 0.431 [c] |
| **CK elevated (>170/ 200 U/l)** [a] | 40 (64.5%) | 62 | 0.493 [d] | **0.014** [e] | 1.000 [e] |
| **TNI$^{max}$ (pg/ml)** | 18.7 (10.6-89.3) | 61 | 0.943 [c] | **0.002** [c] | 0.154 [c] |
| **TNI elevated (>24 pg/ml)** | 27 (44.3%) | 61 | 0.355 [d] | **<0.0001** [d] | 0.689 [e] |
| **Creatinine$^{max}$ (mg/dl)** | 1.2 (0.93-2.1) | 64 | 0.079 [c] | **0.001** [c] | **0.005** [c] |
| **Creatinine elevated (>1.02/ 1.18 mg/dl)** [a] | 38 (59.4%) | 64 | **0.050** [d] | 0.088 [e] | **0.035** [e] |
| **Hemoglobin$^{min}$ (g/dl)** | 10.3 (8.0-12.5) | 65 | 0.941 [c] | **<0.0001** [c] | 0.061 [c] |
| **Hemoglobin decreased (<12/13.5 g/dl)** [a] | 52 (80%) | 65 | 0.218 [d] | 0.071 [e] | 0.329 [e] |
| **Platelet count$^{min}$ /nl** | 178 (134-227) | 65 | 0.950 [c] | 0.232 [c] | **0.001** [c] |
| **Platelet count (<150/nl)** | 22 (33.8%) | 65 | 0.404 [d] | 0.858 [d] | **0.039** [e] |
| **Platelet count (<50/nl)** | 3 (4.6%) | 65 | 1.000 [e] | 1.000 [e] | **0.010** [e] |
| **Absolute leukocytes$^{max}$ /nl** | 7.6 (5.4-9.7) | 62 | 0.381 [c] | 0.069 [c] | 0.647 [c] |
| **Absolute neutrophiles$^{max}$ /nl** | 5.7 (3.5-8.7) | 62 | 0.240 [b] | 0.258 [b] | 0.350 [b] |
| **Absolute lymphocytes$^{min}$ /nl** | 0.7 (0.5-1.0) | 62 | 0.161 [c] | 0.304 [c] | 0.346 [c] |
| **CRP$^{max}$ (mg/l)** | 172 (83-285) | 65 | 0.527 [c] | **<0.0001** [c] | **0.020** [c] |
| **CRP elevated (>5 mg/l)** | 64 (98.5%) | 65 | 0.396 [e] | n.d. | 1.000 [e] |
| **PCT$^{max}$ (ng/ml)** | 0.18 (0.05-1.20) | 65 | 0.166 [c] | **<0.0001** [c] | **0.004** [c] |
| **PCT elevated (>0.5 ng/ml)** | 22 (33.8%) | 65 | 0.381 [e] | **<0.0001** [d] | **0.005** [e] |
| **INR$^{max}$** | 1.2 (1.1-1.5) | 65 | 0.491 [c] | **<0.0001** [c] | **0.001** [c] |
| **INR elevated (>1.25)** | 32 (49.2%) | 65 | 0.968 [d] | **<0.0001** [e] | **0.005** [e] |
| **Fibrinogen$^{max}$ (mg/dl)** | 586 (476-759) | 58 | 0.333 [c] | **<0.0001** [c] | 0.372 [c] |
| **Fibrinogen$^{min}$ (mg/dl)** | 465 (358-531) | 58 | 0.179 [c] | 0.858 [c] | 0.194 [c] |
| **Fibrinogen decreased (<100 mg/dl)** | 0 (0.0%) | 58 | n.d. | n.d. | n.d. |
| **D-dimer$^{max}$ (mg/l)** | 1.67 (0.86-5.08) | 65 | 0.566 [c] | **<0.0001** [c] | **0.001** [c] |
| **D-dimer elevated (>0.5 mg/l)** | 55 (84.6%) | 65 | 0.453 [e] | 0.280 [e] | 0.580 [e] |
| **D-dimer elevated (>2 mg/l)** | 25 (39.7%) | 65 | 0.737 [e] | **0.001** [d] | **0.013** [e] |

Maximum (max) or minimum (min) laboratory values of each patient during the follow up until 15th May 2020 presented as medians (interquartile ranges). Corresponding number and rates (%) of values above (elevated) or below (decreased) the limits of normal or indicated cut-offs are provided as indicated.
CK, creatine kinase; COVID-19, coronavirus disease-2019; CRP, C-reactive protein; DIC, disseminated intravascular coagulopathy; AST, aspartate-aminotransferase, ALT, alanine-aminotransferase; GGT, gamma-glutamyltransferase; INR, international normalized ratio; LDH, lactate dehydrogenase; n.d., not determined; PCT, procalcitonin; TNI, troponin I.

[#]Number of total patients for whom laboratory values were available.

[a] Reference values in brackets for women and men are indicated.

[b] t-test ,[c] Mann-Whitney-U-test, [d] Chi-squared test, [e] Fishers exact test.

**Table S3:** Detailed laboratory data of the seven COVID-19 patients with overt DIC (DIC score ≥5). + Patients who died from COVID-19 during hospitalization.

| **Pat.ID** | **Order date** | **Fibrinogen (mg/dl)** | **Subscore points** | **INR** | **Subscore points** | **Platelet count (/nl)** | **Subscore points** | **D-dimer (mg/l)** | **Subscore points** | **DIC Score total** |
|---|---|---|---|---|---|---|---|---|---|---|
| 13 | 08.04.2020 | 660 | 0 | 2.1 | 2 | 509 | 0 | 6.98 | 3 | 5 |
| 24+ | 31.03.2020 | 316 | 0 | 1.9 | 2 | 93 | 1 | 1.62 | 2 | 5 |
| 50 | 08.04.2020 | 442 | 0 | 1.3 | 1 | 6 | 2 | 5.09 | 3 | 6 |
| 61 | 21.04.2020 | 533 | 0 | 2.8 | 2 | 208 | 0 | 2.24 | 3 | 5 |
| 74 | 08.05.2020 | 834 | 0 | 1.3 | 1 | 65 | 1 | 6.02 | 3 | 5 |
| 79+ | 15.05.2020 | 251 | 0 | 2.2 | 2 | 49 | 2 | 43.72 | 3 | 7 |
| 81+ | 08.05.2020 | 215 | 0 | 3.3 | 2 | 160 | 0 | 40.24 | 3 | 5 |

DIC, disseminated intravascular coagulopathy; INR, international normalized ratio; PatID, patient identification number.
